# Immunotherapy for Urothelial Carcinoma: Current Status and Perspectives

**DOI:** 10.3390/cancers3033055

**Published:** 2011-07-29

**Authors:** Hiroshi Kitamura, Taiji Tsukamoto

**Affiliations:** Department of Urology, Sapporo Medical University School of Medicine, South 1 West 16, Chuo-ku, Sapporo 060-8543, Japan; E-Mail: taijit@sapmed.ac.jp

**Keywords:** immunotherapy, urothelial cancer, cancer vaccine

## Abstract

Intravesical instillation of bacillus Calmette Guérin (BCG) for the treatment of urothelial carcinoma (UC) of the bladder is based on the BCG-induced immune response, which eradicates and prevents bladder cancer. The results of recent studies have suggested that not only major histocompatibility complex (MHC)-nonrestricted immune cells such as natural killer cells, macrophages, neutrophils, *etc.*, but also MHC-restricted CD8^+^ T cells play an important role and are one of the main effectors in this therapy. Better understanding of the mechanism of BCG immunotherapy supports the idea that active immunotherapy through its augmented T cell response can have great potential for the treatment of advanced UC. In this review, progress in immunotherapy for UC is discussed based on data from basic, translational and clinical studies. We also review the escape mechanism of cancer cells from the immune system, and down-regulation of MHC class I molecules.

## 1. Introduction

Since the first report of successful treatment by Morales and associates [1], bacillus Calmette-Guérin (BCG) immunotherapy has been the recommended standard treatment for high grade non-muscle-invasive bladder cancer (NMIBC) [2]. The results of recent studies have suggested that not only innate immunity but also acquired immunity plays an important role in this therapy. In contrast, cisplatin-based chemotherapy, e.g., MVAC (methotrexate/vinblastine/doxorubicin/cisplatin), GC (gemcitabine/cisplatin), *etc.* is a standard systemic therapy for muscle-invasive or metastatic bladder cancer, since urothelial cancer (UC) is chemosensitive. MVAC and GC treatments both result in prolonged survival of up to 14.8 and 13.8 months, respectively, also with long-term followup [3-6]. Several meta-analyses have demonstrated that neoadjuvant cisplatin-containing combination chemotherapy improves overall survival by 5%–7% at 5 years, irrespective of the type of definitive treatment used, whereas neither randomized trials nor meta-analyses have provided sufficient data to support the routine use of adjuvant chemotherapy [7-10]. However, up to 50% of patients are unfit for cisplatin-containing chemotherapy, either due to poor performance status and/or impaired renal function, or to comorbidity that prohibits high-volume hydration [11,12]. Non-platinum combination chemotherapy has produced substantial responses in first- and second-line use, but has not been tested against standard chemotherapy in fit patients or in a purely unfit patient group. There are two main currents for a new paradigm in immunotherapy for UC: (1) enhancement of BCG nonspecific immunotherapy with an adjuvant immunomodulator, e.g., interferon (IFN), granulocyte macrophage colony-stimulating factor (GM-CSF) or specific vaccination for patients with NMIBC, and (2) cancer-specific activation of T cells for patients with muscle-invasive or metastatic UC, e.g., peptide vaccination and dendritic vaccination. In this review, we focus on the progress in immunotherapy for UC and the escape mechanism of cancer cells from the immune system based on data from basic, translational and clinical studies, as well as the down-regulation of major histocompatibility complex (MHC) class I molecules.

## 2. BCG Immunotherapy

### 2.1. Mechanism of BCG Immunotherapy

After intravesical instillation, BCG infects and is internalized into urothelial and bladder cancer cells via a fibronectin-dependent process mediated by integrins [13-16]. Fibronectin attachment protein (FAP) mediates BCG attachment to bladder cancer cells and the bladder wall following intravesical instillation. Recently Sinn *et al.* [17] reported that mice pre-immunized with FAP displayed a significant reduction in tumor growth as a result of BCG therapy, suggesting that FAP was also an effective antitumor agent. The interaction of BCG with urothelial cells is thought to result in several immunologically important changes, including induction of chemokines such as interleukin (IL)-1, IL-6, IL-8, IL-17 [18], GM-CSF, tumor necrosis factor (TNF), and the upregulation of intracellular adhesion molecule (ICAM)-1 [19,20]. These cytokines are considered to prepare the ground for cellular assault by causing tumor cells to display molecules that serve as attachment anchors for immune cells, including neutrophils and T lymphocytes, and activation signals such as ICAM-1, fatty-acid synthetase (FAS), CD40, *etc* [19,21,22]. A high level of IL-8 production is associated with better clinical responses to BCG [23,24].

After several instillations of BCG, various kinds of immune cells such as neutrophils, macrophages, natural killer (NK) cells, T lymphocytes, and NKT cells are recruited. Seventy-five percent of such immune cells, which are contained in the voided urine of bladder cancer patients after BCG therapy, are composed of neutrophils, followed by 5% to 10% macrophages, and 1% to 3% NK cells [25]. The neutrophils are thought to secrete large amounts of cytokines activating various effector cells. Induction of ICAM-1, MHC class I and II molecules on tumor cells is also important to eliminate these cells in this immunotherapy. It takes five or six BCG instillations to induce these immune reactions and a clinical response [26,27].

**Figure 1.**
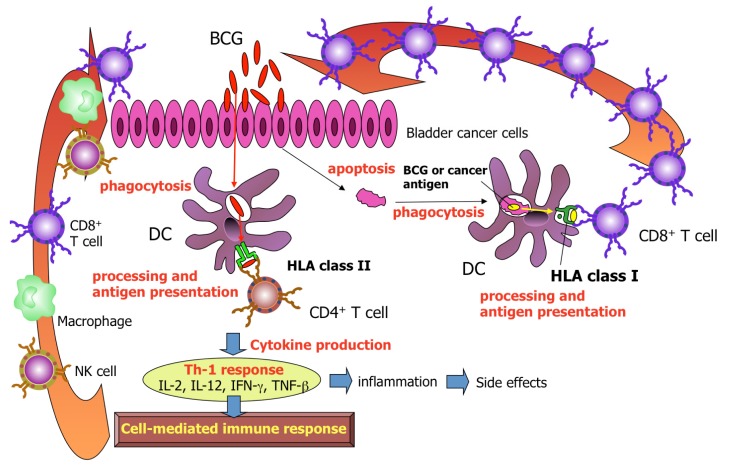
Suggested cascade of immune response induced by intravesical BCG instillation.

Potential effector cells responsible for tumor killing include MHC-nonrestricted cells such as NK cells [28-30], lymphokine-activated killer (LAK) cells [28,31], BCG-activated killer cells [32-34], CD-1-restricted CD8^+^ T cells,[35] γδ T cells [36-38], NKT cells [37-39], neutrophils [40,41], macrophages [42-44], and MHC-restricted CD8^+^ and CD4^+^ T cells [45-48]. Of these cells, T lymphocytes are considered to be the most effective effector cells responsible for eliminating cancer cells [49]. Professional antigen-presenting cells such as dendritic cells (DCs) and macrophages can capture, process and present not only mycobacteria but also antigens from apoptotic cancer cells to T lymphocytes (Figure 1). In a depletion study, both CD8^+^ and CD4^+^ T cells were found to be essential for the successful antitumor effects of BCG [50]. In our clinical data, the good responders had remarkable infiltration of CD8^+^ cells after BCG therapy [47].

Macrophages play important roles not only in antigen presentation but also cytotoxicity. BCG infection may result in up-regulated expression of adhesion molecules such as lymphocyte function-associated antigen-1 (LFA-1) or apoptosis-inducing molecules such as Fas ligand and tumor necrosis factor-related apoptosis-inducing ligand (TRAIL) on macrophages [51-53]. Macrophages can bind to bladder cancer cells via these surface proteins and kill them [48]. Th1 cytokines such as IFN-γ, IL-12, and IL-18 play positive roles in BCG-induced macrophage cytotoxicity toward bladder cancer cells [54,55]. In contrast, Ayari *et al.* [56] reported that high levels of tumor infiltration by CD83^+^ tumor-infiltrating DCs and CD68^+^ tumor-associated macrophages prior to BCG therapy were associated with an increased risk of recurrence. These results may be explained by a switch from a favorable Th1 response of DCs in patients exposed to few BCG instillations to a less favorable Th2 response on repeated BCG instillation [56]. Furthermore, results from some experimental models indicate that BCG therapy induces upregulation of IL-17 and its receptors, and that IL-17 promotes the growth of bladder cancer cells [57,58]. Thus it is suggested that there is a relative predominance of Th1 and Th17 responses and a relative suppression of Th2 and regulatory T cells [49]. However, a recent randomized study showed that autologous intravesical macrophage cell therapy (BEXIDEM®) failed to demonstrate non-inferiority in recurrence rates to intravesical BCG therapy [59]. This result suggests that macrophages from peripheral blood may not elicit antitumor effects as powerful as the immunity induced by BCG instillation.

Chemokines induced by BCG, including IL-8, IL-18, interferon-inducible protein (IP)-10, monocyte chemotactic protein (MCP)-1, MCP-3, macrophage inflammatory protein (MIP)-1α, and MIP-1β, can recruit various immune cells [24,60-63]. There are several reports in which urinary chemokine secretion after intravesical BCG instillation correlated with the clinical response [23,24,62,64,65]. BCG activates innate components of the immune system through toll-like receptor (TLR) 2, TLR4 [66], and TLR9 [67] and induces IFN-γ production in human DCs via TLR2 [68]. BCG can also induce TRAIL expression on polymorphonuclear neutrophils [69]. High TRAIL protein levels in the urine of bladder cancer patients treated with BCG are associated with a favorable response to treatment [70].

### 2.2. Clinical Evidence of Intravesical BCG Therapy

There is no doubt that BCG immunotherapy is an effective therapeutic option for patients with NMIBC. Many prospective randomized studies and meta-analyses have demonstrated that adjuvant BCG therapy after transurethral resection (TUR) for TaT1 disease reduces the risk of recurrence and/or increases the disease-free interval compared with TUR alone or TUR with intravesical chemotherapy [71-78]. Intravesical BCG also reduces the risk of short and long-term treatment failure in patients with carcinoma in situ (CIS), as determined by meta-analysis [79].

Based on the results of meta-analyses [72,75,80,81], BCG therapy given on a maintenance schedule is recommended for patients with intermediate and high risks of progression [82]. Of the randomized trials [83-87] for maintenance BCG therapy, however, only one study [86] demonstrated a benefit for recurrence-free survival of maintenance therapy. It is possible that more patients included in the positive study contributed to the positive results in those meta-analyses. Furthermore, there is no consensus on the protocol for the maintenance schedule, e.g., every 1, 3 or 6 months, or for 1, 2 or 3 years.

The latest European Association of Urology (EAU) guidelines on NMIBC propose the following recommendations: (1) in patients with TaT1 tumors at intermediate or high risk of recurrence and intermediate risk of progression, one intermediate instillation of chemotherapy should be followed by a minimum 1 year BCG treatment or by further instillations of chemotherapy (grade A); (2) in patients at high risk for tumor progression, intravesical BCG for at least 1 year is recommended (grade A), and (3) in patients with bladder CIS, intravesical BCG is recommended for at least 1 year (grade A) [82]. Thus BCG immunotherapy is the mainstay for adjuvant therapy for patients with NMIBC. However, there is a limitation in the efficacy and tolerance of BCG therapy. Although intravesical BCG provides a complete response in 55% to 65% of TaT1 and 70% to 75% of CIS [88,89], 30% fail to respond to BCG or relapse within the first 5 years after treatment and nearly 90% recur by 15 years [88-90]. Furthermore, approximately 20% of patients are BCG intolerant and unable to complete treatment due to side effects [91]. BCG toxicity is not responsible for an improved outcome [92]. Thus there is a need for new immunotherapies providing better outcomes with less toxicity than conventional intravesical BCG therapy.

### 2.3. Immunomodulator of BCG

As mentioned above, intravesical BCG instillation can elicit cytotoxic immune reactions against bladder cancer cells through both innate and adoptive immunity. To augment antitumor responses and limit side effects, combination of BCG and immunomodulating agents such as IFN-α has been evaluated. In a multicenter phase II trial of combination BCG plus IFN-α2b, the 2-year recurrence-free survival rates were 59% and 45% in patients naïve to BCG and those having BCG failure, respectively [93]. A large randomized study of BCG vs. BCG plus IFN-α2b in BCG-naïve patients with NMIBC revealed that BCG plus IFN-α2b did not decrease tumor recurrence and increased the prevalence of side effects compared to BCG alone [94]. To date, there have been no randomized prospective studies that have evaluated IFN-α2b in cases of BCG failure [94]. Another randomized study of mitomycin C followed by BCG vs. BCG plus IFN-α2b showed that no benefit was obtained by alternating IFN-α2b with BCG [95]. Thus addition of IFN-α to BCG does not seem to enhance the antitumor effects of BCG immunotherapy.

## 3. Active Immunotherapy for Advanced UC

Active immunotherapy has the potential to stimulate the immune system, particularly cellular adaptive immunity of the patient to attack the tumor cells. To successfully become recognized as malignant, the response has to be strengthened by vaccination strategies so that target cells can be recognized and eliminated effectively by effector cells such as cytotoxic T lymphocytes (CTLs) [96]. Tumor-infiltrating lymphocytes (TILs) in patients with melanoma, colon cancer and ovarian cancer are associated with favorable clinical outcomes [97]. Several studies of breast cancer indicated that immunological processes, including the development of TILs and disappearance of regulatory T cells, may influence the clinical response to neoadjuvant chemotherapy [98,99]. Lipponen *et al.* [100] investigated the prognostic value of TILs in a cohort of 514 patients with bladder cancer, and reported that a large density of TILs predicted an unfavorable prognosis in papillary tumors, whereas it was a sign of good prognosis in nodular and T3-T4 tumors. Sharma *et al.* [101] showed that patients with T2-T4 disease and higher numbers of CD8^+^ TILs had better disease-free survival and overall survival than those with similar-stage bladder cancer and fewer intratumoral CD8^+^ TILs. Furthermore, Marits *et al.* [102] demonstrated that the sentinel node lymphocytes from patients who underwent radical cystectomy for bladder cancer displayed a retained immunological function upon *in vitro* restimulation with the tumor antigen, and proposed the use of those lymphocytes as the primary source of immune effector cells for immunotherapy. These data support the idea that active immunotherapy through the augmented T-cell response can have great potential for the treatment of advanced bladder cancer.

Active immunotherapeutic strategies include cancer vaccinations using autologous or allogeneic whole tumor cells or tumor lysates, tumor-derived peptides, gene-modified autologous or allogeneic tumor cells, viral vectors carrying genes encoding for tumor antigens, naked DNA plasmids encoding for tumor antigens, autologous APCs presenting tumor antigens, *etc* [103]. To date, however, there have been few phase I or II clinical trials of active immunotherapy for bladder cancer published (Table 1).

Sharma *et al.* evaluated the safety and immunogenicity of a recombinant NY-ESO-1 protein vaccine, which was administered with GM-CSF and BCG as immunologic adjuvants, in a cohort of UC patients whose cancer cells expressed NY-ESO-1, a cancer/testis antigen [104]. Most of the patients exhibited NY-ESO-1-specific antibody and CD4^+^ T cell responses, but CD8^+^ T cell responses were observed in one patient. All patients tolerated the vaccine therapy well and completed all scheduled administrations without any difficulties, although one patient had a grade 2 injection-site reaction consisting of induration and erythema. Honma *et al.* [105] reported the results of a phase I study of survivin peptide vaccination for advanced UC. HLA-A24-restricted survivin-2B peptide was subcutaneously administered with incomplete Freund's adjuvant. Survivin, a member of the inhibitor of apoptosis protein family, is overexpressed in 88% of bladder cancers, but not in normal adult tissues [106]. This vaccination targeting survivin-expressing cells induced epitope-specific CTLs in five of nine patients, and provided tumor reduction and long-term stable disease in two patients. The vaccination was well tolerated in all patients without severe adverse events (AEs). Sherif *et al.* [107] harvested T cells from the nearest draining nodes at cystectomy, and reinfused the autologous T-helper cells after *in vitro* culture and expansion. Tumor-specific-T-helper cells were obtained and reinfused in six of 12 patients without major AEs, but the clinical benefit has not yet been evaluated. Malmström *et al.* [108] carried out a phase I/II study of intravesical administration of adenoviral vectors expressing a CD40 ligand (AdCD40L). CD40L is a potent Th1 immune stimulator that elicits a robust antitumor response. They reported that gene transfer was detected in biopsies, bladders were heavily infiltrated with T cells, and that therapy was well tolerated with low-grade local pain alone. This study suggests that local AdCD40L therapy is potentially effective for bladder cancer, but there are no data on clinical outcomes. Matsumoto *et al.* [109] performed a phase I study of personalized peptide vaccination. Cocktail peptides that might induce HLA-A24-restricted or HLA-A2-restricted and tumor-specific CTL activity were administered subcutaneously. Epitope-specific T cell and antibody responses were observed in eight of 10 patients with one complete response, one partial response and two cases of stable disease. The vaccination was well tolerated with no severe AEs.

The results of the phase I and I/II clinical trials suggest that active immunotherapy for UC induces immune and antitumor responses without severe AEs. However, further clinical studies including phases II and III are needed to determine whether such therapies really provide clinical benefits for patients with UC.

## 4. Escape Mechanism of Cancer Cells from the Immune System in UC

The rationale for studies of active immunotherapy is supported by strong cellular immune responses when introducing cancer-specific CTLs from patients. Immune cells must be activated at the tumor site to manifest appropriate effector mechanisms such as direct lysis or cytokine secretion capable of causing tumor destruction, in addition to the need for a sufficient number of the cells. However, there have been many cases in which no clinical response was achieved regardless of good immune responses in immunomonitoring assays. Therefore, tumor cell factors should be evaluated for development of the clinical responses in cancer immunotherapy.

Human leukocyte antigen (HLA), MHC in humans, class I has a critical role in the recognition and lysis of tumor cells by CTLs, and defects in antigen presentation could allow tumors to escape killing by CTLs [110]. Down-regulation of HLA class I in cancer cells is disadvantageous for presentation of a cancer antigen and its peptide to the patient's immune system [111]. HLA class I is down-regulated in approximately 30% of bladder cancers [106,112]. This is a significant prognostic factor in patients undergoing BCG immunotherapy [47] and radical cystectomy for treatment of muscle-invasive disease [112]. It is suggested that tumor cells with down-regulated HLA class I escape from T cell recognition and that, in such cases, BCG immunotherapy is not effective for patients with bladder cancer. In an animal model using chemically induced bladder cancer, the cytotoxic activity of T cells from rats on tumor cells with low MHC class I expression was decreased as compared with tumor cells with high MHC-I expression [113]. Another recent study using surgical specimens of human bladder cancer demonstrated the changes in HLA class I expression during tumor development [114]. This suggests that immunotherapy stimulates an additional strong cycle of immunoselection, eliminating cells with low HLA and/or changes in it, but cancer cells with more profound HLA class I alterations can escape the immune system and develop recurrent tumors. Thus, it is essential not only to analyze the mechanisms of escape from the immune system but also to develop an immunotherapeutic approach targeting the inhibition of the escape, e.g., upregulation of HLA class I on cancer cells [115].

Regulatory T cells (Tregs) are a population of T cells that regulate the activation of both effector/helper T and B cells. Many studies with cancer patients have demonstrated that the prevalence of Tregs is significantly high in cancerous lesions as compared to healthy controls, and the percentage of Tregs among TILs positively correlates with a significantly lower survival rate [116]. In contrast, Winerdal *et al.* [117] reported that the infiltration of Tregs in bladder cancer was also associated with improved prognosis. Thus the immune escape mechanism through Treg is still controversial in bladder cancer.

Myeloid-derived suppressor cells (MDSCs) represent a heterogeneous population of immunosuppressive cells expressing a variety of surface markers such as CD11c^+^, CD11b^+^, CD33^+^, CD34^+^ and CD15^+^. The frequency of these cells also positively correlates with the incidence of recurrence or metastatic disease in patients [116]. Eruslanov *et al.* [118] have demonstrated that highly activated inflammatory myeloid cells inhibiting *in vitro* T cell proliferation through induction of Tregs in peripheral blood and tumor tissues from patients with bladder cancer represent a source of multiple chemokines/cytokines and may contribute to inflammation and immune dysfunction in bladder cancer. Further studies are needed to confirm these results, examine the underlying mechanisms in detail, and investigate the prognostic impact on survival.

## 5. Conclusions and Perspectives

Various studies have indicated that immunotherapy can eliminate bladder cancer cells, but it is not yet capable of curing bladder cancer, and other therapeutic modalities such as surgery and chemotherapy are still required for this purpose. BCG immunotherapy is effective for NMIBC but does not always control its recurrence and is ineffective for MIBC, indicating that BCG therapy may not induce powerful specific antitumor immunity or immunological memory. However, based on the efficacy and recent advances in understanding of the mechanism of BCG immunotherapy, active immunotherapy has great potential as a weapon against even advanced urothelial carcinoma. Although an HLA class I-restricted peptide vaccine eliciting epitope-specific CTLs seems to be one of the most promising approaches at present, this can also induce CD8^+^ T cell tolerance [119,120], with low response rates in clinical trials. Better understanding of the innate and adaptive immune responses, and of the immune escape mechanisms underlying immunological tolerance, and acknowledgment of the importance of adaptive immunity for control of tumor growth are necessary to develop a more comprehensive immunotherapeutic approach [121]. Alternatively, combination with chemotherapy, molecular-targeting therapy or surgery can be effective ways to apply active immunotherapy to clinical use.

## Figures and Tables

**Table 1.** Overview of phase I and II clinical trials of active immunotherapy in bladder cancer.

**Author**	**Approach**	**Disease stage**	**No. Pt**	**Phase**	**Results**
Sharma *et al.* (2008) [104]	NY-ESO-1 protein vaccine + BCG i.d. + GM-CSF	Radically operated UC without metastasis (adjuvant setting)	6	I	Ag-specific Ab in 5/6 Pts, CD8 T cell response in 1/6 Pts, CD4 T cell response in 6/6 Pts, no severe AE
Honma *et al.* (2009) [105]	Survivin peptide vaccine	Refractory recurrent UC	9	I	CD8 T cell response in 5/9 Pts, tumor reduction in 2/9 Pts, no severe AE
Sherif *et al.* (2010) [107]	Reinfusion of autologous T-helper cells	T2-T4 N1-2 M0-1 bladder cancer	12	I	Feasible in 6/12 Pts, technical failure in 6/12 Pts, no severe AE
Malmström *et al.* (2010) [108]	Adenoviral vector expressing CD40 ligand (intravesical)	MIBC scheduled cystectomy (phase I), Ta disease (phase II)	8	I/II	Enhancement of T cell infiltration and IFN-γ production, reduction of circulating Treg, AE of minor local pain
Matsumoto *et al.* (2010) [109]	Personalized peptide vaccine	Advanced UC (MVAC failure)	10	I	1 CR, 1 PR, 2 SD, PFS 3.0 months, OS 8.9 months, no severe AE

i.d.: intradermal; Ag: antigen; Ab antibody; AE: adverse event; Treg: regulatory T cell; MVAC: methotrexate, vinblastine, adriamycin and cisplatin; CR: complete response; PR: partial response; SD: stable disease; PFS: progression-free survival; OS: overall survival
